# 造血干细胞体外扩增及应用研究进展

**DOI:** 10.3760/cma.j.issn.0253-2727.2022.02.018

**Published:** 2022-02

**Authors:** 清 李, 莎 郝, 涛 程

**Affiliations:** 中国医学科学院血液病医院（中国医学科学院血液学研究所），实验血液学国家重点实验室，国家血液系统疾病临床研究中心，细胞生态海河实验室，天津 300020 State Key Laboratory of Experimental Hematology, National Clinical Research Center for Blood Diseases, Haihe Laboratory of Cell Ecosystem, Institute of Hematology & Blood Disease Hospital, Chinese Academy of Medical Sciences & Peking Union Medical College, Tianjin 300020, China

造血干细胞（hematopoietic stem cell, HSC）是一类成体组织干细胞，通过定向分化产生淋系和髓系等造血祖细胞，进而大量增殖分化成各类成熟的功能性血细胞以维持整个机体的造血稳态。HSC具有自我更新、多系分化、凋亡、运动迁徙和保持静息等生物学特性。正是由于HSC独特的生物学特性，其被用于治疗多种疾病，如血液系统良恶性疾病、自身免疫性疾病（如黏多糖贮积症Ⅱ型）及实体肿瘤（如视神经母细胞瘤）等[1]–[2]。同时，随着基因生物学的基础研究不断向临床转化，干细胞基因治疗也已成为当今研究的一个主流热点，如应用HSC基因疗法可治疗Fanconi贫血、β-地中海贫血及镰状细胞病等遗传性血液病[3]–[5]。

与单倍型造血干细胞移植（haplo-HSCT）相比，脐血干细胞移植（UCBT）具有脐血资源充足、便于采集、对HLA错配的耐受性高、造血干细胞和造血祖细胞集落形成潜能高等优点[6]–[7]，且移植后移植物抗宿主病（GVHD）发生率和复发率较低、患者生存质量较好[8]–[9]。因此，UCBT在临床上逐渐成为治疗血液系统恶性疾病的主要治疗方法之一。但干细胞数量不足限制了UCBT在临床上的广泛应用。有研究表明，双份脐血干细胞移植（dUCBT）可弥补干细胞数量不足的缺陷，但由于双份脐血来源于不同的个体，移植物间存在竞争关系，最终在患者体内只能存留优势供者来源细胞[10]–[11]。

因此，无论是UCBT的推广还是干细胞基因治疗的临床转化，都亟需解决HSC扩增这一根本性问题。目前，用于干细胞扩增的技术和方法层出不穷，为UCBT和干细胞基因治疗的实际应用提供了条件。本文就HSC体外扩增方法以及扩增干细胞的应用进展进行综述，为后续进一步研究HSC体外扩增方法及其临床转化奠定基础。

一、造血干细胞体外扩增

（一）应用造血刺激因子实现HSC体外扩增

细胞因子是首批用于HSC体外扩增的药物之一。多项研究表明细胞因子可在体内影响小鼠HSC数量。小鼠骨髓分析结果表明所有长期造血干细胞（LT-HSC）均表达成纤维细胞生长因子（FGF）受体，在HSC与小鼠成骨细胞共培养的无血清培养基中添加FGF1和FGF2可支持HSC离体扩增和再生[12]。FGF受体衍生物已用于支持培养基中短期造血干细胞（ST-HSC）和LT-HSC的扩增及存活[13]。用含细胞因子SCF、Flt3L、G-CSF、IL-3、IL-6的培养基对人脐血CD34^+^CD38^−^细胞培养4 d后，脐血CD34^+^CD38^−^细胞扩增了4倍，同时集落形成单位（CFU）数量增加了10倍，NOD/SCID小鼠中SCID-再生细胞（NOD-SCID repopulating cells, SRC）增加了2～4倍[14]。另一项研究表明，人脐血CD34^+^CD38^−^细胞在含有Flt-3、SCF、IL-3、IL-6和G-CSF的无血清培养基中培养5～8 d，CFU可扩增100倍，长期培养起始细胞（LTC-IC）增加4倍，竞争性再生单位（CRU）增加2倍[15]。

血小板生成素（TPO）作为巨核细胞的增殖和分化因子，在HSC生物学中也具有重要作用。Yagi等[16]研究表明，TPO在LT-HSC和ST-HSC中均诱导自我更新和扩增。另有研究显示，单独或与IL-3、FLT3L、SCF和IL-6等细胞因子组合，TPO在体外具有诱导HSC增殖的作用，促进移植后造血重建[17]–[18]。此外，TPO通过其受体c-MPL的信号传导在维持HSC的功能中起关键作用。NR101是c-MPL激动剂，通过与c-MPL结合，诱导c-MPL下游JAK2、STAT5、STAT3等信号传导途径激活，可有效增加CD34^+^细胞的数量[19]。

血管生成素样蛋白（angiopoietin-like proteins, ANGPTL）包括糖基化的分泌蛋白，其在代谢、炎症、癌症及造血中起重要作用。不同的ANGPTL均参与刺激小鼠骨髓中HSC的扩增。数据表明，在无血清的STIF培养基（SCF、TPO、IGF-2、FGF-1）中培养高纯度的小鼠HSC 10 d后，HSC数量增加约8倍[20]，而在加入ANGPTL2或ANGPTL3共培养后，其数量扩增约20倍，长期移植后CRU数量增加24～30倍，植入率则增加了30～52倍[21]。其他亚型的ANGPTL如ANGPTL4、5、7也同样具有扩增HSC的作用[21]。以上研究结果表明ANGPTL于小鼠HSC的扩增和存活是必需的。

Notch配体Jagged或Delta样配体通过与细胞上的Notch受体结合从而激活Notch信号通路。Notch信号传导途径是HSC命运决定和淋巴细胞生成的重要途径，人造血干祖细胞（HSPC）中Notch信号通路的异常激活可促进HSPC的自我更新。DELTA1^ext-IgG^（DXI）是一种工程化的Delta样配体，由Delta1的细胞外结构域和人免疫球蛋白1（IgG1）的Fc部分融合而成。使用DXI激活人脐血CD34^+^细胞中的Notch信号通路可实现具有临床意义的ST-HSPC体外扩增，同时缩短移植后中性粒细胞植入时间[22]–[23]。在低氧条件下，利用DXI对人G-CSF动员外周血（G-CSF mobilized peripheral blood, mPB）来源的CD34^+^细胞进行体外培养21 d后，功能性LT-HSC的数量扩增了4.9倍。Araki等[24]认为这与低氧刺激可减轻体外培养过程中产生的内质网应激反应、减少ROS产生以及减少与增殖相关的细胞凋亡等细胞应激性损伤有关。此外，Notch信号通路的激活可进一步促进多效生长因子（pleiotrophin）诱导的HSC体外扩增[25]。

TNFSF15是肿瘤坏死因子超家族的成员之一，主要参与维持血管内稳态。Ding等[26]发现TNFSF15在体外也具有扩增HSC的作用。利用IMDM培养基（SCF、TPO、Flt3L）联合TNFSF15对人脐血CD34^+^细胞进行体外培养，7 d后LT-HSC数量明显扩增，SRC数量增加了3.14倍。机制研究表明，TNFSF15是通过激活Notch信号通路来促进人脐血来源HSC进行体外扩增。

Wnt是一类分泌性脂蛋白，可与frizzele受体结合。Wnt信号传导是一种在进化过程中高度保守的信号转导途径，根据对β-连环蛋白的依赖性可分为经典wnt信号通路和非经典wnt信号通路。这两种wnt信号转导途径在调节HSC中都发挥着至关重要的作用，主要与维持HSC处于静息状态相关[27]。Wnt信号传导主要以剂量依赖性方式调控造血过程[28]。经典wnt信号参与大部分成体干细胞系统的自我更新过程，对体外HSC的维持也是必要的。研究表明，高水平的经典Wnt信号导致HSC干性丢失，促进HSC分化[29]。Duinhouwer等[30]证明外源wnt3a蛋白能够抑制脐血来源的CD34^+^细胞在无血清培养基中的扩增作用，降低多谱系CFU-GEMM的数量及扩增HSPC的长期重建能力。

前列腺素E2（PGE2）是哺乳动物细胞中最活跃的前列腺素，参与增殖和凋亡等多种过程。有研究表明，PGE2能刺激静止的骨髓细胞进入周期循环、增殖和分化[31]。利用PGE2对小鼠HSC进行体外培养，2 h后HSC数量显著增加[32]。PGE2还可增强脐血HSC归巢基因（CXCR4）、增殖基因（cyclinD1）和存活基因的表达。

（二）利用化合物对HSC进行体外扩增

1. 小分子化合物：

（1）糖原合成酶激酶-3（Gsk-3）抑制剂和mTOR抑制剂：在小鼠HSC中，Gsk-3参与Wnt和mTOR两条通路，前者主要与促进HSC自我更新相关，后者参与HSC的谱系分化作用。用Gsk-3β抑制剂6-bromoindirubin 3′-oxime（BIO）抑制Gsk-3的作用，可有效促进HSPC的体外扩增和长期造血重建，同时能增强HSC早期植入[33]–[34]。结节硬化症复合体（TSC）-mTOR信号通路是调控细胞代谢的重要通路，可通过抑制线粒体生物功能和活性氧物质（ROS）维持HSC的功能和静息状态。利用mTOR抑制剂rapamycin对小鼠HSC进行体外培养，通过抑制HSC的衰老有效扩增HSC，并能有效增强HSC在体内的长期造血重建能力[35]。在添加了GSK-3抑制剂CHIR99021和mTOR抑制剂rapamycin（CR）的无血清、无细胞因子的培养基中培养小鼠LSK细胞，7 d后HSC数量增多且具备长期造血重建能力；该体系也同样适用于培养人脐血来源的CD34^+^细胞，体外培养第3天，总细胞数增加了约7倍且保留长期造血重建能力[36]。

（2）嘌呤衍生物StemRegenin 1（SR1）：芳香烃受体（aryl hydrocarbon receptor, AHR）是一个碱性螺旋-环-螺旋同源域蛋白的转录因子，属于bHLH超家族，在HSC命运决定中起重要作用[37]。SR1是AHR的拮抗剂，仅作用于人HSC，而不影响小鼠HSC。研究表明，SR1可使人脐血CD34^+^细胞在离体环境下扩增50倍，其中具有长期造血重建能力的LT-HSC数量增加约17倍[38]。此外，SR1能选择性促进人胚胎干细胞来源多能干祖细胞的扩增[39]。AHR拮抗剂可增加表达CXCR4蛋白的HSC数量，参与归巢过程，促进HSC的植入[40]–[41]。

（3）嘧啶吲哚衍生物类似物UM171：通过高通量筛选化学库，Fares等首次发现了一种嘧啶吲哚衍生物类似物UM171，该化合物可在体外显著扩增脐血来源的HSC[42]，还可诱导多能干细胞向HSPC分化[43]。此外，UM171处理使LT-HSC数量增加约13倍，联合使用UM171与SR1对HSC进行体外培养可实现细胞倍数显著增加[42]。研究表明，UM171主要通过调节HSC炎症信号通路和抑制ROS积累来实现HSC的体外扩增[44]–[45]。利用细胞表面蛋白ITGA3可有效富集出UM171诱导的扩增HSC中具有长期造血重建功能的LT-HSC，有助于提高培养HSC临床应用的成功率[46]。

（4）聚乙烯醇（PVA）：PVA是一种有机化合物，主要用于制造胶水、乳化剂、粘合剂和分散剂等。之前，PVA主要用于胚胎干细胞的培养研究[47]。一项最新研究发现，PVA可用于HSC的长期离体扩增[48]–[49]。一直以来，血清白蛋白都被认为是HSC培养过程中生物污染的主要来源，Wilkinson等[49]在无血清培养体系中加入PVA对小鼠HSC进行长期离体培养，1个月后发现功能性HSC扩增了236～899倍，具有长期造血重建能力的HSC扩增了54～204倍。在此研究基础上有望大幅降低HSC的培养成本，从而改善临床上用于HSC移植治疗HSC不足的问题。

（5）细胞周期抑制剂：哺乳动物的细胞周期进程主要受细胞周期蛋白cyclins、细胞周期蛋白依赖性激酶CDK和细胞周期蛋白依赖性激酶抑制蛋白CDKI复合物调控。其中，CDKI可通过抑制cyclins和CDK来抑制HSC的细胞分裂，以维持HSC的静息状态。激活HSC并促使其进入细胞周期是目前HSC体外扩增研究的关键。Albayrak等[50]利用S期激酶相关蛋白2（SKP2）抑制剂SKP2-C25增加HSC的细胞周期活性，使人CD34^+^细胞得到明显扩增。在培养基中使用5-氮杂-2-脱氧胞苷（5aza-2-deoxycytidine, 5azaD）和曲古抑菌素A（TSA）作为染色质重塑剂，CD34^+^CD90^+^ HSC扩增了12倍，与自我更新过程相关基因（HOXB4、BMi1和GATA2）表达上调，而C-MYC等细胞周期基因表达下调[51]–[52]。在INK4蛋白家族中，P18（INK4C）在G1期的细胞周期调节中具有重要作用。P18IN003和P18IN011是P18的小分子合成抑制剂。研究表明，使用这些抑制剂对LSK细胞处理后细胞数量增加了近4倍。培养16周后，HSC的植入能力增强[53]。小分子化合物005A是P18的另一种小分子抑制剂，用该化合物处理人脐血CD34^+^细胞使得功能性HSC数量增加2.72倍，005A可能是通过延缓细胞分裂，同时激活Notch信号通路和HoxB4转录因子表达，从而导致LT-HSC的自我更新能力增强[54]。

（6）表观遗传修饰对HSC自我更新和分化的影响：Mikkola等[55]发现，MLLT3可通过结合转录活性位点及组蛋白H3K79me2发挥其维持造血干细胞自我更新能力的功能，过表达MLLT3使LT-HSC在体外扩增约12倍。组蛋白去乙酰化酶3是HSC扩增的重要因子，其抑制作用可促进HSC体外扩增[56]。Garcinol作为组蛋白乙酰转移酶的非特异性抑制剂，通过抑制赖氨酸382（K382）上的P53乙酰化使得人脐血HSC的水平升高[57]。丙戊酸和维生素B5是高特异性和强效的组蛋白去乙酰化酶（HDAC）抑制剂，通过抑制HDAC使人脐血HSC数量扩增，HSC上CD90、CD117、CD49F、CXCR4和HOXB4的表达增强，醛脱氢酶活性增加，同时改善HSC的归巢能力[58]–[61]。

2. 无机化合物：盐离子（铁、钙、铜、镁和锌等）在调节细胞增殖、分化等代谢功能方面具有重要作用。据报道，培养基中大量的铜离子可促进HSC分化，其螯合剂四亚乙基五胺（TEPA，也称为StemEx）可通过螯合培养基中的铜离子来有效抑制HSC分化进而促进其增殖。Peled等[62]发现，利用IL-6、TPO、SCF、FL和TEPA富集的培养基对CD133^+^ HSC进行培养，其数量扩增了89倍，该方法有效突破了体外扩增HSC过程中早期干细胞分化耗尽的瓶颈。用TEPA短期培养人HSC可以增强这些细胞在NOD-SCID小鼠中的重建能力。Luchsinger等[63]研究表明，通过降低HSC细胞内钙离子水平来抑制钙蛋白酶活性可以稳定TET2，从而有助于体外HSC功能的维持。而利用钙离子通道阻滞剂SKF来抑制钙池操纵性钙内流（SOCE）可通过增加CaR/CasR、CXCR4和黏附分子等细胞表面分子的表达量来诱导人CD34^+^ HSC的体外扩增，并促进HSC的归巢和植入[64]。

一氧化氮（NO）是一种半衰期短且极不稳定的生物自由基，共有三种同型。同型Ⅰ具有钙离子依赖性，而同种型Ⅱ（iNOS）不依赖于钙离子，同型Ⅲ由内皮细胞产生。其中，同型Ⅱ可使CD34^+^ HSC在与HL60单核细胞共培养的环境中分化增加[65]。Reykdal等[66]也证明，用iNOS抑制剂L-NIL培养CD34^+^ HSC，7 d后CD34^+^HSC的数量扩增了13.4倍，同时有效减少细胞凋亡。

（三）使用骨髓微环境细胞辅助HSC体外扩增

1. HSC命运受到骨髓微环境细胞的调节：间充质干细胞（MSC）作为骨髓微环境的一类重要细胞，通过与微环境中其他细胞之间进行相互作用和分泌细胞因子来调控HSC的命运。临床试验结果显示，用与MSC共培养的HSC进行移植是安全有效的。与接受双份未处理的脐血移植的患者相比，接受与MSC共培养的单份脐血联合单份未处理脐血移植的患者造血植入得到明显改善，中性粒细胞和血小板恢复时间更短[67]。Huilin等[68]证实利用工程化表达腺病毒E4orf1基因（hFLSECs-E4orf1）的人胎肝窦内皮细胞与脐血CD34^+^HSC共培养使CD34^+^细胞数量扩增3.15倍。Luo等[69]研究显示，M2巨噬细胞与脐血CD34^+^ HSC共培养导致CD34^+^细胞数量增加3.8倍。Orticelli等[70]利用人羊膜MSC与人脐血CD34^+^细胞共培养7 d后，LT-HSC扩增3倍，ST-HSC扩增33倍，同时HSC的自我更新潜能和长期造血重建能力得以维持。Nakahara等[71]研究表明，利用慢病毒载体转染KOXII（Klf7、Ostf1、Xbp1、Irf3、Irf7）至体外培养的MSC中，可使其重获功能；将重编程的MSC（rMSC）与人脐血CD34^+^细胞共培养6 d后，功能性LT-HSC的细胞数量增加了7倍；机制研究表明，rMSC主要通过减轻体外扩增HSC的DNA损伤和复制压力来对HSC进行有效的体外扩增。

2. 微载体与HSC扩增：Bai等[72]利用一种两性离子材料构成的三维水凝胶包裹HSC的方法对HSC进行体外扩增，通过模拟HSC体内骨髓微环境实现长期稳定的体外扩增，同时保持HSC的再生潜能。利用该方法对人脐血CD34^+^ HSC培养24 d后，LT-HSC扩增了近73倍。该研究对干细胞治疗领域的进展具有重要意义。

二、HSC扩增的临床应用

（一）造血干细胞移植

造血干细胞移植（HSCT）是目前最经典且成熟的干细胞临床应用方案，是某些血液系统及非血液系统疾病的唯一治愈方法。然而，供者不足仍然是HSCT在临床应用上的主要限制因素，对于allo-HSCT尤其如此。随着HSC体外扩增技术的逐渐成熟，由基础研究向临床应用转化也有相关报导[73]–[75]。用16-二甲基前列腺素E2处理的16个脐血样本的Ⅰ期临床试验研究结果表明，与对照组相比，接受该治疗的患者的中性粒细胞恢复加快，同时证实了其安全性[73]。SR1处理使CD34^+^细胞扩增330倍，与接受普通UCBT患者相比，接受经SR1扩增的脐血CD34^+^细胞移植的白血病患者，中性粒细胞和血小板植入时间明显缩短，且无植入失败病例[74]。一项加拿大的早期临床试验表明，接受UM171扩增处理的UCBT移植患者，移植后中性粒细胞植入的中位时间为9.5 d，血小板恢复的中位时间为42 d，且无移植失败病例和意外不良事件发生。该研究证实了利用UM171扩增处理的脐血HSC进行UCBT是安全可行的[76]。尽管已有相关临床研究数据表明经体外扩增的人CD34^+^ HSC是安全可靠的，然而在体外培养过程中维持HSC自我更新和扩增的最佳培养体系及相关分子通路等至今尚未明确，对于体外培养的HSC广泛应用于临床还需进一步的深入探索。

（二）造血干细胞基因治疗

使用allo-HSCT来治疗遗传性血细胞疾病已成为临床有效途径之一，但受到合适的供者和潜在的免疫并发症的限制。因此，使用自体HSC基因疗法可以避免这些局限。随着基因编辑对患者自体HSC进行遗传校正的技术逐步改进，越来越多的疾病有望被成功治愈。基因治疗成功率与HSC的纯化程度有关，HSC越纯，干性越强，基因治疗成功率越高。正因如此，自体HSC基因疗法也一定程度上受到HSC数量不足的限制，通过离体扩增患者自体HSC，在一定程度上可增加基因治疗的成功率。Zonari等[77]分别用慢病毒载体转染纯化的人脐血CD34^+^和人骨髓 CD34^+^细胞，用SR1分别对转染后的CD34^+^细胞进行体外培养7～12 d，应用流式细胞术分选出CD34^+^ CD90^+^和CD34^+^ CD90^−^细胞后植入NSG小鼠体内，结果显示CD34^+^CD90^+^细胞具有长期造血重建能力。随后利用不同浓度梯度的SR1和UM171单一或联合用药对来自mPB、慢病毒载体转染的CD34^+^CD38^−^ HSC和祖细胞（HPSC）进行体外扩增，发现二者联合培养对HSPC的扩增作用具有累积效应，且培养后的转染HSPC植入率更高，SRC比例也更高。该研究表明离体扩增进行基因修饰后的HSPC是可行且有效的，但在临床转化上需进一步优化方案和开展更深入的研究。

三、总结与展望

过去十年的研究表明HSC离体扩增实际上是可行的。在对HSC自身调节以及调节体内HSC自我更新的微环境特异性因子更深入理解的基础上，新的离体扩增HSC方法陆续出现。然而，许多微环境中的HSC调控机制仍然未能阐明，并且需要对这些方法进行广泛而有力的验证，这是基础向临床转化必要的第一步。在不久的将来，HSC扩增无疑将出现多种模式、策略和方法，在干细胞研究和临床应用中这无疑是值得期待的。
